# Supplementary material for: Iterative improvement in the automatic modular design of robot swarms
Source: PeerJ Comput Sci. 2020 Dec 7;6:e322. doi: 10.7717/peerj-cs.322 (PMC7924708; doi:10.7717/peerj-cs.322)
Supplement: Supplemental Information 3 [file peerj-cs-06-322-s003.zip › argos3/doc/api/standalone/a00581_source.html]

ARGoS: plugins/simulator/entities/omnidirectional\_camera\_equipped\_entity.h Source File


- Main Page
- Related Pages
- Namespaces
- Classes
- Files

- File List
- File Members

# plugins/simulator/entities/omnidirectional\_camera\_equipped\_entity.h

Go to the documentation of this file.

```
00001 
00007 #ifndef OMNIDIRECTIONAL_CAMERA_EQUIPPED_ENTITY_H
00008 #define OMNIDIRECTIONAL_CAMERA_EQUIPPED_ENTITY_H
00009 
00010 namespace argos {
00011    class COmnidirectionalCameraEquippedEntity;
00012    class CEmbodiedEntity;
00013 }
00014 
00015 #include <argos3/core/simulator/entity/entity.h>
00016 #include <argos3/core/utility/math/vector3.h>
00017 #include <argos3/core/utility/math/quaternion.h>
00018 
00019 namespace argos {
00020 
00021    class COmnidirectionalCameraEquippedEntity : public CEntity {
00022 
00023    public:
00024 
00025       ENABLE_VTABLE();
00026 
00027    public:
00028 
00034       COmnidirectionalCameraEquippedEntity(CComposableEntity* pc_parent);
00035 
00046       COmnidirectionalCameraEquippedEntity(CComposableEntity* pc_parent,
00047                                            const std::string& str_id,
00048                                            const CRadians& c_aperture,
00049                                            const CVector3& c_offset);
00050 
00055       virtual void Init(TConfigurationNode& t_tree);
00056 
00061       inline const CVector3& GetOffset() const {
00062          return m_cOffset;
00063       }
00064 
00069       inline void SetOffset(const CVector3& c_offset) {
00070          m_cOffset = c_offset;
00071       }
00072 
00077       inline const CRadians& GetAperture() const {
00078          return m_cAperture;
00079       }
00080 
00085       inline void SetAperture(const CRadians& c_aperture) {
00086          m_cAperture = c_aperture;
00087       }
00088 
00089       virtual std::string GetTypeDescription() const {
00090          return "omnidirectional_camera";
00091       }
00092 
00093    private:
00094 
00096       CRadians m_cAperture;
00097 
00099       CVector3 m_cOffset;
00100 
00101    };
00102 }
00103 
00104 #endif
```

---

Generated on 10 Jul 2018 for ARGoS by 
 1.6.1
